# Portomesenteric Venous Thrombosis in an Emergency Department Patient After Laparoscopic Sleeve Gastrectomy

**DOI:** 10.7759/cureus.19872

**Published:** 2021-11-24

**Authors:** Connor Lawler, Briana King, Melody L Milliron

**Affiliations:** 1 Emergency Medicine, Lake Erie College of Osteopathic Medicine, Erie, USA; 2 Emergency Medicine, Allegheny Health Network, Erie, USA

**Keywords:** mesenteric venous thrombosis, sleeve gastrectomy, laparoscopic, venous thrombosis, portomesenteric

## Abstract

Postoperative abdominal pain after gastric surgery requires thorough evaluation in the ED. Portomesenteric venous thrombosis (PMVT) is a rare complication after laparoscopic sleeve gastrectomy, which requires prompt evaluation and diagnosis. Patients require admission with prompt anticoagulation and broad-spectrum antibiotics due to the risk of decompensation from intestinal ischemia and sepsis from bowel translocation. This report describes the case of a 36-year-old male who presented to the ED one week after laparoscopic sleeve gastrectomy with tachycardia and gradual onset, severe, sharp epigastric abdominal pain associated with anorexia and fatigue. He subsequently developed hypotension requiring vasopressor support, acute kidney injury, thrombocytopenia, and septic shock suspected due to secondary to bowel translocation. He was transferred to another facility for consideration for thrombolysis and went on to recover. This case report describes a rare case of PMVT after laparoscopic sleeve gastrectomy. Surgical risk factors include obesity and multiple components of Virchow’s triad. These include inherited/acquired thrombophilic states, iatrogenic endothelial injury of portal vein/mesenteric vessels via direct manipulation, and increased intraabdominal pressure decreasing portal venous flow. Providers should carefully consider evaluation for genetic hypercoagulability requiring lifelong anticoagulation. On hospital discharge, anticoagulation should continue for at least six months, with repeat CT with IV contrast or USG in three to six months to evaluate for recanalization of the venous system. Knowledge of the appropriate evaluation and treatment of this rare complication after laparoscopic sleeve gastrectomy is vital to avoid unnecessary patient morbidity and mortality.

## Introduction

Portal venous thrombosis (PVT) refers to occlusion of the portal vein by a thrombus. Portomesenteric venous thrombosis (PMVT) occurs when thrombus extends into the mesenteric venous system and commonly includes the superior mesenteric vein and splenic vein [1]. The most common cause of PVT is cirrhosis [2]. Other causes include prothrombotic states during active malignancy, abdominal trauma, and prothrombotic conditions such as primary myeloproliferative disorders [2,3,4], as well as inherited prothrombotic disorders such as factor V Leiden mutation, antithrombin III deficiency, protein C and S deficiency, G20210A prothrombin mutation, homocystinuria, and chronic inflammatory conditions of the abdominal viscera (pancreatitis, liver abscess, inflammatory bowel disease) [2,3,4]. Acute PMVT is an uncommon but potentially life-threatening complication of laparoscopic surgery. We describe a case of acute PMVT diagnosed in an emergency department patient after laparoscopic gastric sleeve gastrectomy. We review diagnosis and ED treatment plan to prevent complications such as mesenteric ischemia and infarction. We also discuss initiation of broad-spectrum antibiotics to prevent bowel translocation and ED anticoagulation with consideration to evaluation further for underlying coagulopathy.

## Case presentation

**Figure 1:**
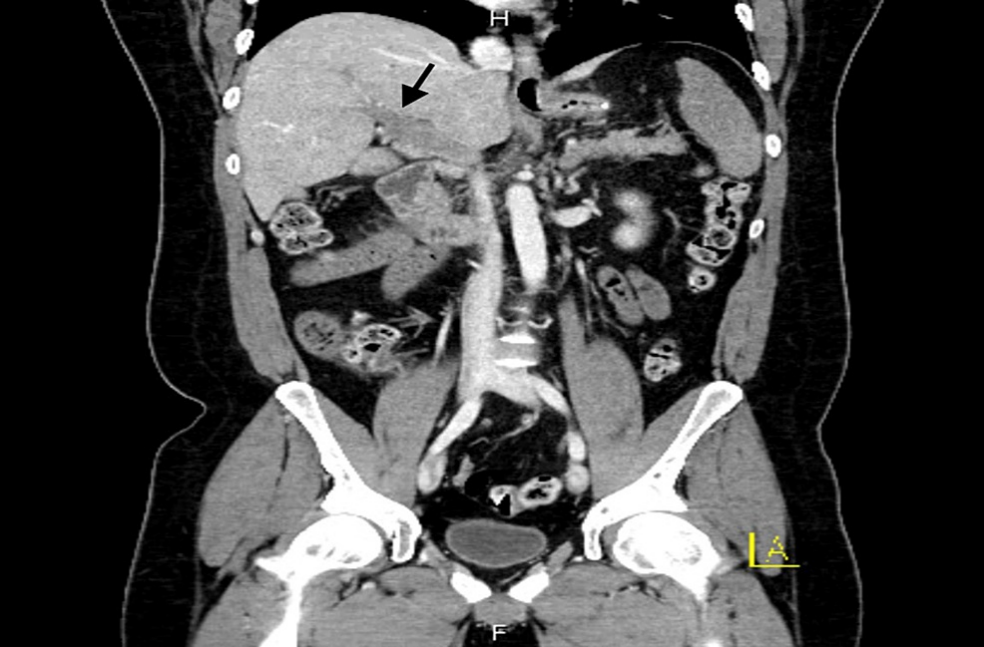
CT with contrast coronal images show nonenhancement of portal venous system consistent with complete thrombosis of portal venous system (black arrow).

**Figure 2:**
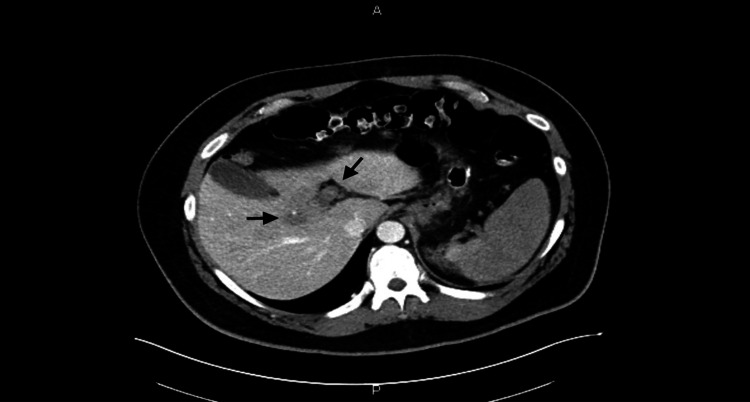
CT with contrast transverse images show nonenhancement of portal venous system consistent with complete thrombosis of portal venous system (Black arrows).

A 36-year-old male presented to the ED one week after laparoscopic sleeve gastrectomy with chief complaint of gradual onset, severe, sharp epigastric abdominal pain associated with anorexia and fatigue. Initial physical exam revealed a mildly dehydrated and uncomfortable appearing male with sinus tachycardia in the 130s and a nonacute abdomen with epigastric tenderness without rigidity, rebound, or guarding. Surgical sites were well healing, clean, dry, intact and without drainage. CT of the abdomen and pelvis with intravenous (IV) contrast was performed (Figure 1 and Figure 2), initially reviewed by the ED physician and was discussed with reading radiologist.

CT report detailed nonenhancement of the splenic vein with complete thrombosis of the portal venous system including main, right, and left porta veins, splenic vein with extension into the superior mesenteric vein and associated branches, with possible internal hernia following surgery. USG imaging redemonstrated thrombosis of the portal venous system. General Surgery and Vascular Surgery departments recommended continued medical management. Our patient required ICU admission on heparin drip, empiric piperacillin-tazobactam for enteric pathogen coverage, and IV hydration. He subsequently developed hypotension requiring vasopressor support, acute kidney injury, thrombocytopenia, and septic shock suspected due to secondary to bowel translocation. Our patient was transferred to an outside facility for consideration of thrombolysis, which was not performed due to patient improvement. The patient went on to recover and was discharged home on Eliquis® (Pfizer Inc., New York) for six months with repeat CT scan in three to six months to evaluate for recanalization of PMVT.

## Discussion

Although rare after laparoscopic procedures, PVT occurs due to components of Virchow’s triad. Procoagulopathic factors include inherited/acquired thrombophilic states, iatrogenic endothelial injury of portal vein/mesenteric vessels via direct manipulation, and increased intraabdominal pressure decreasing portal venous flow [4,5]. While undergoing laparoscopic sleeve gastrectomy, intraabdominal pressure increases due to insufflation. Increased intraabdominal pressure compresses the liver vasculature, portal vein, mesenteric veins, and splenic veins, resulting in a decrease in splenic and small intestinal blood flow and overall venous stasis [1,4,5]. Other risk factors include reverse Trendelenburg positioning during surgery, retained carbon dioxide causing increased portal venous pressure, dehydration, and improper duration of prophylactic anticoagulation [1,5]. In addition to other comorbidities, morbid obesity leads to a systemic predisposition for thrombophilia [1]. A thrombus may form in the portal vein, splenic vein, or mesenteric veins and cause venous congestion, which may lead to intestinal ischemia, portal hypertension, and septic PVT [1,2].

While PVT is common during certain procedures such as splenectomy and liver transplantation, secondary to direct manipulation of the portomesenteric vasculature, PVT is a rare event after sleeve gastrectomy [1,4,5]. In a systemic literature review, James et al. (2009) reported an increased risk of PVT after Roux-en-Y gastric bypass, which is a more invasive procedure than sleeve gastrectomy [5]. Hence, consideration of exclusion of inherited or acquired thrombophilic conditions is warranted in a patient with PVT after sleeve gastrectomy [5].

The diagnosis of PVT is challenging due to vague clinical manifestations such as non-specific abdominal pain, nausea, vomiting, fever, ascites, or larger than expected wound drainage [4]. If PVT extends into the mesenteric vessels it may result in ileus, hematochezia, fever, leukocytosis, elevated liver function tests, and sepsis. Symptoms typically manifest one to three weeks postoperatively but vary from three to 42 days postoperatively [5].

Physical examinations were normal in seven out of 18 patients with PVT [5]. As a result, imaging is key in the evaluation of PVT. USG may show hypoechoic material in the vessel lumen and increased diameter of the portal vein and tributaries [3], and color doppler may enhance the accuracy of diagnosis [3,4]. However, USG may be limited due to poor visualization of the mesenteric vessels and portal venous system. In these cases, CT with IV contrast or magnetic resonance angiography is necessary [3]. Although less timely in the ED setting, magnetic resonance imaging or angiography has a sensitivity and specificity of 98% and 100% respectively [2].

Treatment goals in PVT and PMVT include recanalization of thrombosed vasculature, prevention of recurrence, and treatment of complications [1-5]. Patients with PVT related to laparoscopic surgery should be anticoagulated with subcutaneous low-molecular-weight heparin or intravenous heparin if invasive procedures are under consideration and then transitioned to oral anticoagulants for six months [3-5]. In the setting of genetic predisposition to thrombophilia, lifelong anticoagulation is recommended [1,3,5]. Systemic thrombolytic therapy is reserved for patients with severe or refractory disease due to significant bleeding risk [3]. Surgical thrombectomy increases morbidity and mortality compared to mechanical or aspiration thrombectomy [3]. In cases of bowel ischemia, exploratory laparotomy may be necessary [1]. Prognosis is patient-dependent, with a worse prognosis for those that develop bowel ischemia or sepsis [2,3]. In follow-up, routine Doppler USG or CT with IV contrast should be performed at three and six-month intervals to analyze for recanalization [3]. Partial or complete recanalization is achieved in a large majority of patients with acute PVT treated solely with anticoagulation [1].

## Conclusions

PMVT is a rare complication after laparoscopic sleeve gastrectomy, which requires prompt evaluation and diagnosis. Physical exam may be normal and evaluation by CT with IV contrast or USG with doppler is crucial. Patients require admission with prompt anticoagulation and broad-spectrum antibiotics due to the risk of decompensation from intestinal ischemia and sepsis from bowel translocation. Providers should carefully consider evaluation for genetic hypercoagulability requiring lifelong anticoagulation. On hospital discharge, anticoagulation should continue for at least six months, with repeat CT with IV contrast or USG in three to six months to evaluate for recanalization of the venous system. Knowledge of the appropriate evaluation and treatment of this rare complication after laparoscopic sleeve gastrectomy is vital to avoid unnecessary patient morbidity and mortality.
